# Lipid Disorders and Cardiovascular Risk: A Comprehensive Analysis of Current Perspectives

**DOI:** 10.7759/cureus.51395

**Published:** 2023-12-31

**Authors:** Maha Wazir, Olusegun A Olanrewaju, Muhammad Yahya, Jaya Kumari, Narendar Kumar, Jagjeet Singh, Abbas Yasir Abbas Al-itbi, Komal Kumari, Aqsa Ahmed, Tamur Islam, Giustino Varrassi, Mahima Khatri, Satesh Kumar, Hina Wazir, Syed S Raza

**Affiliations:** 1 Department of Medicine, Khyber Teaching Hospital, Peshawar, PAK; 2 Department of Pure and Applied Biology, Ladoke Akintola University of Technology, Ogbomoso, NGA; 3 Department of General Medicine, Stavropol State Medical University, Stavropol, RUS; 4 Department of Medicine, Liaquat University of Medical and Health Sciences, Jamshoro, PAK; 5 Department of Internal Medicine, Mehran Medical Centre, Karachi, PAK; 6 Department of Internal Medicine, Burjeel Hospital, Abu Dhabi, ARE; 7 Department of Internal Medicine, Lahore General Hospital, Lahore, PAK; 8 Department of Medicine, Jordan University of Science and Technology (JUST), Irbid, JOR; 9 Department of Medicine, NMC Royal Family Medical Centre, Abu Dhabi, ARE; 10 Department of Medicine, Medicare Hospital, Faisalabad, PAK; 11 Department of Internal Medicine, Allied Hospital, Faisalabad, PAK; 12 Department of Pain Medicine, Paolo Procacci Foundation, Rome, ITA; 13 Department of Internal Medicine/Cardiology, Dow University of Health Sciences, Karachi, PAK; 14 Department of Medicine, Shaheed Mohtarma Benazir Bhutto Medical College, Karachi, PAK; 15 Department of Internal Medicine, Khyber Medical College, Peshawar, PAK; 16 Department of Physiology, Gajju Khan Medical College, Swabi, PAK; 17 Department of Physiology, Khyber Medical College, Peshawar, PAK; 18 Robert and Suzanne Tomsich Department of Cardiothoracic Surgery, Cleveland Clinic Florida, Peshawar, PAK; 19 Department of Physiology, Gandhara University, Peshawar, PAK

**Keywords:** precision medicine in cardiovascular health, lipoprotein subtypes, dyslipidemia, atherosclerosis, cardiovascular risk, lipid disorders

## Abstract

The increasing worldwide prevalence of cardiovascular diseases (CVDs) highlights the need to understand the complex relationships between lipid abnormalities and elevated cardiovascular risk. This review thoroughly investigates the complex terrain of lipid abnormalities, highlighting their crucial significance in developing CVDs. Dyslipidemia, which is closely connected to atherosclerosis, is a significant risk factor for CVDs, including coronary artery disease, myocardial infarction, and stroke. This review thoroughly examines the intricate relationship between lipoproteins, cholesterol metabolism, and the inflammatory cascade, providing a detailed comprehension of the mechanisms that contribute to atherogenic processes. An extensive analysis of the occurrence and distribution of lipid diseases worldwide indicates a concerning high frequency, which calls for a reassessment of public health approaches. Dyslipidemia is caused by a combination of genetic predispositions, lifestyle factors, and metabolic abnormalities, as supported by significant data. Moreover, investigating different types of lipoproteins and their specific functions in the development of atherosclerosis provides insight into the complex causes of CVDs. In addition to conventional lipid profiles, newly identified biomarkers and advanced imaging techniques are being carefully examined for their ability to improve risk classification and treatment strategies' effectiveness. From a critical perspective, the review thoroughly examines the current state of lipid-modifying medicines, specifically statins, fibrates, and new therapeutic approaches. The text discusses the emerging concept of precision medicine, which involves tailoring treatment approaches to individuals based on their genetic and molecular characteristics. This approach has the potential to improve treatment outcomes. In addition, this study critically assesses the effects of lifestyle changes and nutritional interventions on lipid homeostasis, offering a comprehensive view of preventive strategies. This review consolidates current viewpoints on lipid diseases and their complex correlation with cardiovascular risk. This review contributes to the ongoing cardiovascular disease prevention and management dialogue by clarifying the molecular mechanisms, exploring new therapeutic options, and considering broader societal implications.

## Introduction and background

Cardiovascular diseases (CVDs) are a significant worldwide health problem that demands attention because of their widespread effects on illness and death rates. According to the World Health Organization, CVDs are the primary cause of death worldwide, resulting in almost 17.9 million deaths per year [1]. The complex origins of CVDs involve a sophisticated interaction between genetic, environmental, and lifestyle variables. Among these factors, dyslipidemia plays a crucial role in the development of CVDs. The extensive impact of CVDs worldwide necessitates a thorough analysis to understand its immense scope fully. According to the most recent data, the occurrence of CVDs is increasing, which presents a significant public health problem in many populations and locations. The economic consequences of CVDs are similarly substantial, as healthcare systems globally struggle with the increasing expenses linked to the prevention, diagnosis, and treatment of these conditions [1]. The figures highlight the immediate necessity for a thorough comprehension of the risk factors linked to CVDs, specifically focusing on lipid disorders. The increase in CVDs is closely connected to the aging population, urbanization, and lifestyle changes. The shift from infectious to non-infectious diseases in low- and middle-income nations exacerbates the impact of cardiovascular disorders. Changes in eating habits, inactive lifestyles, and an increasing occurrence of obesity drive the transition. These factors all contribute to the worldwide impact of lipid disorders and, as a result, CVDs. There is a significant link between lipid problems and the development of CVDs, including atherosclerosis, which is the primary pathological process responsible for most cardiovascular events. Dyslipidemia refers to a group of lipid abnormalities, which include high levels of low-density lipoprotein cholesterol (LDL-C), low levels of high-density lipoprotein cholesterol (HDL-C), and elevated triglycerides. These lipid abnormalities create the conditions for the development of atherosclerotic plaques in the walls of arteries, which is closely connected to the beginning and advancement of CVDs [2]. Multiple epidemiological research and clinical trials have clearly shown the association between dyslipidemia and adverse cardiovascular outcomes. The renowned Framingham Heart Study began in 1948 and was crucial in clarifying the link between elevated cholesterol levels and coronary heart disease. Further studies, such as the Lipid Research Clinics Coronary Primary Prevention Trial and the Scandinavian Simvastatin Survival Study (4S), have provided additional information on the effectiveness of therapies that lower lipid levels in preventing cardiovascular events [2].

The link between these two factors can be explained by the deposition of lipoproteins that promote atherosclerosis development in the arteries' inner layer. This leads to a series of inflammatory reactions and changes in the structure of the blood vessels [2]. Therefore, it is crucial to comprehend the complicated dynamics of lipid diseases to unravel the intricacies of cardiovascular risk. This extensive study offers a comprehensive overview of current viewpoints on lipid diseases and their significant implications for cardiovascular well-being. We aim to analyze much scientific material to understand the complex connections between dyslipidemia and CVDs. We will focus on the molecular, genetic, and environmental elements that cause these illnesses [3]. When exploring lipid diseases, we will examine the many types of lipoproteins and explain their specific functions in the development of atherosclerosis. This study will examine the range of lipid profiles associated with cardiovascular risk, from the traditional distinction between LDL-C and HDL-C to newer biomarkers like lipoprotein-a (Lp-a). We will focus on understanding the series of inflammatory reactions that occur alongside dyslipidemia and explore the complex pathways that contribute to the advancement of atherosclerosis. Although there have been significant breakthroughs in our comprehension of lipid problems and their association with cardiovascular risk, there still needs to be more information that hinders the development of specific therapy methods and precision medicine techniques. This study aims to identify and assess these deficiencies thoroughly and analytically, with a particular focus on areas that require additional investigation. With the changing environment of cardiovascular health, there are ongoing advancements in biomarkers and imaging techniques, which offer prospects for more precise risk assessment [4]. The incorporation of genetic and molecular profiling into clinical practice has the potential to provide personalized treatment approaches.

Nevertheless, to achieve actual enhancements in patient outcomes, it is necessary to thoroughly assess the current gaps in knowledge and apply these breakthroughs accordingly. This review aims to contribute to the ongoing discussion in cardiovascular medicine by thoroughly examining these aspects. It seeks to guide future research efforts and provide valuable information for evidence-based clinical practice. By synthesizing current perspectives and identifying areas lacking knowledge, this review plays a crucial role in advancing our understanding of lipid disorders and reducing their impact on global cardiovascular health.

## Review

Methodology

The methodology in the narrative review titled "Lipid Disorders and Cardiovascular Risk: A Comprehensive Analysis of Current Perspectives" is carefully designed to integrate current scientific knowledge thoroughly. This review does not require ethics committee approval as it relies solely on previously published data and literature. The study employs a thorough methodology in gathering data from reliable databases such as PubMed, MEDLINE, Web of Science, and other scholarly sources. A wide range of lipid diseases, cardiovascular risks, and related topics are included as keywords to enable a thorough retrieval of relevant papers. This temporal focus aims to encompass the latest discoveries and insights in the field of lipid diseases and cardiovascular risk. The literature's narrative synthesis is structured topically, focusing on essential elements such as lipoprotein subtypes, cholesterol metabolism, inflammatory pathways, and new biomarkers. This systematic method thoroughly investigates the complicated connection between lipid diseases and cardiovascular risk, providing a holistic comprehension for the audience. The methodology incorporates ethical considerations, carefully adhering to academic integrity and acceptable research methods. The process of citing and attributing sources is carried out carefully, guaranteeing proper recognition to the original writers. Moreover, the narrative review method aims to ensure the precise depiction and understanding of the current literature while avoiding any bias or misinterpretation. Nevertheless, it is crucial to recognize the inherent constraints of this methodology. The narrative synthesis, however thorough, depends on the accessibility and comprehensiveness of published data, which may restrict the inclusion of specific results or views. The absence of primary data collection and analysis can limit the depth of insights generated from the review. Additionally, the reliance on previously published data exposes the check to the possibility of inherent biases or omissions in the original studies.

Epidemiology of lipid disorders

CVDs continue to be a major cause of illness and death worldwide, with dyslipidemia playing a significant role in their development. This essay offers a comprehensive analysis of the epidemiology of lipid diseases, investigating the global occurrence of dyslipidemia and the complex interaction between genetic and environmental factors.

Prevalence of Dyslipidemia Worldwide

Dyslipidemia varies significantly across regions, indicating an intricate interaction between genetic, environmental, and sociodemographic elements. Epidemiological studies demonstrate apparent variations between continents, with developed countries frequently displaying elevated rates of dyslipidemia. An extensive examination of worldwide data conducted by the Global Burden of Disease Study revealed that Western Europe and North America exhibit elevated levels of total cholesterol compared to sub-Saharan Africa and South Asia [4]. Numerous factors, such as food patterns, lifestyle preferences, and healthcare infrastructure, shape the geographical disparities. Sociodemographic characteristics are crucial in determining the geographical prevalence of dyslipidemia. The increasing frequency of dyslipidemia in developed nations can be attributed to urbanization, marked by sedentary lifestyles and dietary changes. In contrast, in underdeveloped areas, where traditional eating habits and increased levels of physical exertion are common every day, the prevalence of dyslipidemia may be relatively lower. These geographical subtleties emphasize the significance of adapting public health treatments to the unique difficulties encountered by various communities. In addition to regional differences, sociodemographic factors substantially impact the epidemiology of lipid diseases. Age is a significant factor that influences the prevalence of dyslipidemia, as it tends to increase as people get older. The Framingham Heart Study, a critical long-term examination, revealed a distinct increase in serum cholesterol levels with age, highlighting the significance of age as a separate risk factor for dyslipidemia [5]. Gender differences are apparent, as men frequently have elevated levels of LDL-C and reduced levels of HDL-C compared to women. Furthermore, socioeconomic position has a role in the discrepancies observed in the prevalence of dyslipidemia. Individuals with lower socioeconomic levels may experience hurdles to acquiring appropriate nutritional options, participate in fewer preventative health activities, and encounter challenges in controlling lipid diseases. This socioeconomic gradient is recognized worldwide, surpassing regional limitations and emphasizing the significance of addressing social factors in public health policies aimed at dyslipidemia [5].

Genetic and Environmental Influences

The etiology of dyslipidemia is significantly influenced by genetic factors, which contribute to the observed variations in lipid profiles among different groups. Familial hypercholesterolemia (FH) is a genetic ailment that is characterized by high levels of LDL-C, which is a kind of cholesterol. This condition demonstrates how genetic predispositions can affect how lipids are processed in the body. Research has identified genetic abnormalities impacting the low-density lipoprotein (LDL) receptor gene as significant factors in FH [5]. The heritability of lipid characteristics goes beyond FH and includes the combined effects of multiple genes that determine an individual's vulnerability to dyslipidemia. Twin and family studies have presented convincing data supporting the inheritability of lipid problems. The heritability estimates for total cholesterol, LDL-C, HDL-C, and triglycerides range from 40% to 60%, highlighting the significant influence of genetic factors on the variation in lipid levels. Genome-wide association studies (GWAS) have provided additional clarity on the genetic structure of lipid characteristics, revealing numerous regions of the genome linked to lipid metabolism and the risk of CVD [6]. Genetic predispositions create the foundation for dyslipidemia, whereas lifestyle and metabolic factors serve as changeable factors that can either reduce or worsen the genetic risk. Lifestyle decisions, such as eating patterns and physical exercise, significantly impact lipid profiles. Diets that contain high amounts of saturated fats and trans fats lead to increased levels of LDL-C while consuming more omega-3 fatty acids has been linked to positive alterations in lipid profiles [6]. The Mediterranean diet, defined by a substantial consumption of fruits, vegetables, and unsaturated fats, has consistently correlated with reduced dyslipidemia and cardiovascular events. Physical activity, a fundamental aspect of a healthy way of life, has a double purpose in lipid metabolism. Scientific studies have demonstrated that regular physical activity increases HDL-C levels, which helps transport cholesterol in the opposite direction and protects against the development of atherosclerosis [7]. Conversely, sedentary behaviors and a lack of physical activity contribute to dyslipidemia and enhance cardiovascular risk. The complex interaction between hereditary variables and lifestyle decisions emphasizes the significance of tailored strategies in managing dyslipidemia. Metabolic variables, such as obesity and insulin resistance, regulate lipid balance. Adipose tissue functions as an endocrine organ, secreting adipokines that impact lipid metabolism and systemic inflammation. Obesity, especially the accumulation of fat around the internal organs, is linked to dyslipidemia, characterized by high levels of triglycerides, low levels of HDL-C, and a lipid profile promoting atherosclerosis development. Insulin resistance, a characteristic feature of metabolic syndrome, has a role in dyslipidemia by affecting many processes, such as the regulation of lipolysis and the metabolism of lipids in the liver [8]. Although epidemiological studies offer essential insights into the occurrence and causes of dyslipidemia, it is important to recognize its inherent limitations. Cross-sectional approaches, frequently utilized in epidemiological research, offer a momentary glimpse of relationships but do not definitively demonstrate causality. Longitudinal research and randomized controlled trials are necessary to establish the sequence of events across time and evaluate the effects of interventions on lipid profiles. Furthermore, the use of self-reported data has the risk of recall bias and misclassification, especially when it comes to nutritional assessments. The presence of different measuring procedures and variations in lipid profile thresholds among studies can further impede direct comparisons. Furthermore, the changing criteria for diagnosing dyslipidemia, including modifications in cholesterol standards, require meticulous analysis of patterns over some time. The ethical considerations in epidemiological research on dyslipidemia mainly concern participant permission, confidentiality, and the appropriate sharing of data. In numerous epidemiological studies, participant data is anonymized to protect privacy, while researchers strictly follow ethical norms and gain informed consent from study participants. To uphold the credibility of research findings and public confidence, providing clear and comprehensive documentation of techniques and any potential conflicts of interest is crucial. To summarize, studying lipid diseases provides valuable insights into the worldwide impact of dyslipidemia. Regional differences, sociodemographic factors, genetic predispositions, and lifestyle choices influence the frequency and patterns of dyslipidemia worldwide. It is essential to acknowledge these intricacies to customize efficient public health treatments that tackle the distinct difficulties encountered by varied communities. Genetics establishes the foundation, while lifestyle and metabolic factors are changeable elements that influence outcomes, providing chances for tailored interventions. Recognizing the inherent constraints of epidemiological methods and adhering to ethical principles are crucial for enhancing our comprehension of lipid diseases and improving global cardiovascular risk management strategies.

Lipid metabolism and atherogenic processes

Lipid metabolism plays a crucial function in regulating the balance of different types of lipoproteins and maintaining cholesterol levels in the body. This process is essential in developing atherosclerosis, a significant contributor to cardiovascular illnesses. This essay extensively examines lipid metabolism, focusing on the functions of specific lipoprotein subtypes and the intricate regulation of cholesterol homeostasis.

Classification of Lipoprotein Subtypes

LDLs, commonly called "bad cholesterol," play a crucial role in the development of atherosclerosis. LDL particles transport cholesterol from the liver to peripheral tissues, enabling cellular processes. Nevertheless, when existing in surplus or altered states, LDL becomes a formidable factor in the development of atherosclerosis. The method initiates with the penetration of LDL into the artery intima, where it experiences oxidative alterations. Modified LDL triggers an inflammatory reaction, which attracts monocytes that differentiate into macrophages, commencing the process of foam cell development. The formation of foam cells and the buildup of plaques rich in lipids contribute to the development of atherosclerotic lesions [8]. Multiple studies highlight the correlation between high LDL levels and a heightened risk of CVD. The groundbreaking Framingham Heart Study demonstrated a clear and direct association between elevated LDL cholesterol levels and coronary heart disease. Furthermore, the effectiveness of interventions that lower LDL cholesterol, specifically statins, in reducing cardiovascular events has been demonstrated by clinical trials such as the 4S and the Cholesterol Treatment Trialists' (CTT) Collaboration [8,9]. Understanding the mechanisms that cause atherosclerosis due to LDL cholesterol lays the groundwork for precise therapeutic interventions. The research aims to clarify the elements that affect the vulnerability of LDL to oxidation and the specific biochemical mechanisms that trigger inflammatory responses. Approaches like LDL receptor (LDLR) modification and the creation of anti-inflammatory substances present encouraging possibilities for interrupting the atherogenic process initiated by LDL. In contrast to the atherogenic characteristics of LDL, high-density lipoproteins (HDLs) are recognized as essential components in safeguarding cardiovascular health. HDL, also known as "good cholesterol," aids reverse cholesterol transport by eliminating surplus cholesterol from peripheral tissues and sending it back to the liver for disposal [9]. This process provides anti-atherogenic effects by inhibiting the buildup of cholesterol in the walls of arteries. HDL has a preventive effect against atherosclerosis that goes beyond its role in transporting cholesterol. HDL possesses anti-inflammatory and antioxidant characteristics, which prevent the oxidation of LDL and reduce the inflammatory reactions occurring in the artery wall. In addition, HDL enhances endothelial function and regulates the activity of many enzymes involved in lipid metabolism. The clinical evidence about the cardiovascular advantages of HDL is complex and subtle. Although epidemiological studies have repeatedly shown a negative correlation between HDL levels and cardiovascular risk, attempts to increase HDL levels through therapies have had varying outcomes. The lack of cardiovascular risk reduction in extensive clinical trials, such as the Atherothrombosis Intervention in Metabolic Syndrome With Low HDL/High Triglycerides: Impact on Global Health Outcomes (AIM-HIGH) and Heart Protection Study 2-Treatment of HDL to Reduce the Incidence of Vascular Events (HPS2-THRIVE) investigations, when using pharmacological treatments that target HDL, highlights the intricate nature of HDL biology and emphasizes the necessity for a more detailed comprehension of its roles [9]. The research in this field aims to clarify the many parts of HDL particles, comprehend the variability within the HDL fraction, and pinpoint specific molecular targets that might be utilized for therapeutic objectives. The continuous investigation of therapeutics based on HDL and the possible incorporation of genetic knowledge into HDL metabolism holds the potential to provide new approaches for managing cardiovascular risk.

Regulation of Cholesterol Levels

Cholesterol homeostasis is controlled at the cellular level, maintaining a balance between the intake, release, and production of cholesterol. Cells obtain cholesterol by engaging in receptor-mediated endocytosis of LDL particles facilitated by LDLRs on the cell membrane [9]. The cholesterol absorbed by the cells is either used for cellular processes or retained in cellular compartments. De novo cholesterol production, mainly in the endoplasmic reticulum, is an additional mechanism that helps control cellular cholesterol levels. The enzyme that controls the rate of this process is 3-hydroxy-3-methylglutaryl-coenzyme A reductase (HMG-CoA reductase). Statins, a commonly given medication for high cholesterol, lower cholesterol levels by blocking HMG-CoA reductase, which reduces the production of cholesterol [10]. Cellular homeostasis is maintained through the intricate equilibrium of cholesterol intake, synthesis, and utilization. Disruption of these mechanisms can result in the buildup of cholesterol inside cells, which is a precursor to the development of atherosclerosis. The research focuses on understanding the molecular mechanisms that control cholesterol balance by finding essential regulatory proteins and pathways. The disruption of lipid metabolism is a characteristic feature of dyslipidemia, playing a crucial role in the development and advancement of atherosclerosis. Various factors, such as genetic predispositions and lifestyle decisions, come together to disturb the delicate equilibrium between different types of lipoproteins and the regulation of cholesterol levels. Congenital abnormalities that impact proteins involved in lipid metabolism, such as the LDLR and apolipoprotein B (APOB), can cause FH and significantly increase LDL levels. This makes individuals more susceptible to developing premature atherosclerosis [10]. The selection of one's lifestyle, such as consuming diets rich in saturated fats and engaging in inactive behaviors, worsens dyslipidemia by encouraging the production and buildup of LDL cholesterol. Furthermore, diseases such as insulin resistance and metabolic syndrome contribute to dyslipidemia by creating a favorable environment for the development of atherosclerosis. Insulin resistance disrupts the usual control of lipolysis, resulting in increased triglyceride levels and reduced HDL cholesterol. The metabolic syndrome, a group of defined cardiovascular risk factors, increases the likelihood of atherosclerosis developing by boosting inflammation and oxidative stress [11]. Gaining insight into the molecular mechanisms behind dyslipidemia-related disruptions in lipid metabolism establishes a basis for developing therapeutic therapies. Pharmacological substances that specifically target essential enzymes involved in the production of cholesterol, such as statins, have demonstrated effectiveness in lowering LDL levels and reducing the risk of CVDs [12]. Current research is investigating new targets in the lipid metabolism pathway to create more specific and effective therapies. Ultimately, the complex interaction between different types of lipoproteins and the regulation of cholesterol levels influences the overall functioning of lipid metabolism and the development of atherosclerosis. LDL, due to its ability to promote the development of atherosclerotic plaques, plays a role in the initiation and progression of these lesions. On the other hand, HDL, with its beneficial effects on the heart, works against these processes. Cholesterol homeostasis, meticulously controlled at the cellular level, maintains a precise balance between cholesterol absorption, production, and utilization. Comprehending the subtle distinctions in lipid metabolism forms the basis for specific treatment strategies designed to reduce dyslipidemia and prevent atherosclerosis. Research efforts persist in exploring the many roles of different types of lipoproteins, understanding the intricacies of cholesterol balance, and discovering new molecular targets for drug interventions. As we negotiate this changing environment, the pursuit of precision medicine in lipid management shows potential for customizing therapies according to individual genetic profiles and attaining better cardiovascular risk reduction.

Inflammatory cascade and cardiovascular pathogenesis

The inflammatory cascade is a crucial factor in developing and advancing atherosclerosis, a complex process in cardiovascular pathophysiology. This essay explores the various facets of inflammation, explaining its significant influence on the development of atherosclerosis. We thoroughly investigate the complex relationship between the immune response in atherogenic lesions and the impact of cytokines on endothelial dysfunction.

Immune Response in Atherogenic Lesions

In the development of atherosclerosis, inflammation has transitioned from being a passive observer to an active contributor to the disease's progression. Atherosclerosis, the primary etiology of most cardiovascular disorders, is distinguished by the buildup of lipids, inflammatory cells, and fibrous components within the walls of arteries. Atherosclerosis begins with endothelial dysfunction, which allows circulating monocytes to enter the subendothelial region. Upon entering the artery intima, monocytes differentiate into macrophages, initiating a complex immunological response. Altered LDL plays a crucial role in this process. Macrophage scavenger receptors identify oxidatively changed LDL, leading to the uptake of these altered lipoproteins and the creation of foam cells. Foam cells, which contain lipid droplets, gather together to create fatty streaks, which are the initial observable abnormalities in the development of atherosclerosis [12]. The immune response within atherogenic lesions goes beyond macrophages and involves a range of immune cells. T cells, essential regulators of the adaptive immune response, invade atherosclerotic lesions and engage with macrophages, thereby enhancing the inflammatory process. Research utilizing modern imaging techniques such as intravascular ultrasonography (IVUS) and PET has yielded valuable information about the dynamic interactions between immune cells and atherosclerotic plaques [13]. Moreover, the adaptive immune response stimulates T cells with antigens found in atherogenic lesions. Modified self-proteins, known as autoantigens, boost the immune system to attack the components of the artery wall, leading to persistent inflammation. The interaction between the innate and adaptive immune systems provides an environment that promotes the development of atherosclerosis, leading to its ongoing progression. To devise precise therapeutic approaches, a comprehensive understanding of the subtle distinctions in the immune response within atherogenic lesions is of utmost importance. Emerging techniques aim to regulate the activity of immune cells within plaques, prevent the production of foam cells, and interrupt the communication between various resistant cell types. Using immunomodulatory drugs, like anti-inflammatory biologics, can modify the immunological environment in atherosclerotic lesions and slow down the course of the disease [13].

Cytokines and Endothelial Dysfunction

Cytokines, crucial agents in the immune response, have a key role in coordinating inflammation in the arterial wall. They significantly contribute to endothelial dysfunction, a characteristic feature of early atherosclerosis. Endothelial dysfunction is when there is a disturbance in the normal functioning of blood vessels, resulting in decreased availability of NO and increased activation of pro-inflammatory signaling pathways. Tumor necrosis factor-alpha (TNF-α), a crucial cytokine that promotes inflammation, significantly impacts endothelial cells. TNF-α enhances endothelial activation by increasing the expression of adhesion molecules, including vascular cell adhesion molecule-1 and intercellular adhesion molecule-1. This process enhances the attachment and movement of immune cells into the area underneath the endothelium layer, which plays a role in the beginning of atherosclerosis [14]. Interleukin-1 (IL-1) is a significant cytokine involved in endothelial dysfunction. IL-1 stimulates the production of chemicals that promote inflammation and molecules that facilitate the attachment of cells to the inner lining of blood vessels, intensifying the process of inflammation. Furthermore, IL-1 plays a role in attracting and stimulating immune cells in atherogenic lesions.

Directing efforts towards IL-1 signaling pathways has become a treatment approach, as clinical trials have shown that inhibiting IL-1 can lead to cardiovascular advantages. The complex interaction of cytokines goes beyond TNF-α and IL-1. Interleukin-6 (IL-6), interleukin-12, and interferon-gamma (IFN-γ) are a few of the several cytokines that have a role in the development of atherosclerosis. For example, IL-6 facilitates the transformation of T cells into pro-inflammatory subgroups, which aids in the continuation of long-lasting inflammation within atherosclerotic plaques. IFN-γ, released by activated T cells, amplifies the pro-inflammatory characteristics of macrophages, hence intensifying the inflammatory environment [14]. Endothelial dysfunction, which is controlled by the actions of cytokines, goes beyond inflammation. Reduced levels of nitric oxide (NO), which is a characteristic of endothelial dysfunction, impede the widening of blood vessels and encourage the narrowing of blood vessels, hence leading to hypertension, a significant risk factor for CVD. The complex impacts of cytokines on endothelial function highlight their diverse roles in developing cardiovascular disorders. Studies examining the effects of anti-cytokine treatments, including those aimed at TNF-α and IL-1, have positively impacted cardiovascular health in inflammatory disorders such as rheumatoid arthritis. Nevertheless, applying these discoveries to larger groups of individuals with cardiovascular conditions is still being actively studied. Challenges encompass the diverse effects of cytokines and the requirement for precise regulation of immune responses while maintaining vital physiological functioning.

Biomarkers and advanced imaging in risk stratification

Within the field of cardiovascular risk assessment, the range of available methods has grown beyond conventional lipid profiles to encompass newly identified biomarkers and sophisticated imaging techniques. This essay thoroughly examines the process of categorizing risk levels, examining the intricate details of traditional lipid profiles, and investigating the potential of new biomarkers like Lipoprotein (a), apolipoproteins, and advanced imaging techniques for identifying atherosclerotic lesions.

Conventional Lipid Profiles

Conventional lipid profiles, which include LDL-C and HDL-C, are fundamental in evaluating the risk of CVD. LDL-C, also known as "bad cholesterol," significantly contributes to the development of atherosclerosis. The connection between high levels of LDL-C and an enhanced risk of CVD has been solidly established by the Framingham Heart Study and multiple clinical trials [14]. Consequently, LDL-C continues to be a primary focus for medical treatments, and statins have proven effective in reducing LDL-C levels and decreasing cardiovascular events. In contrast, HDL-C, commonly called "good cholesterol," is linked to a reduced likelihood of experiencing cardiovascular events. HDL particles are involved in reverse cholesterol transport, which consists of removing surplus cholesterol from tissues outside the liver and delivering it to the liver for elimination. Epidemiological studies, such as the Prospective Cardiovascular Münster project, have repeatedly shown a negative association between HDL-C levels and the risk of CVD. Nevertheless, translating this observed connection into successful therapeutic approaches has been difficult, as interventions targeting the increase of HDL-C levels have produced inconsistent outcomes in clinical studies [15]. Triglycerides and total cholesterol, essential elements of the conventional lipid profile, offer supplementary insights in evaluating the risk of CVD. Heightened triglyceride levels are linked to a heightened risk of atherosclerosis, especially when accompanied by illnesses like metabolic syndrome and diabetes. The aggregate of LDL-C, HDL-C, and VLDL-C, known as total cholesterol, offers a complete overview of an individual's lipid profile. Although traditional lipid profiles are essential for assessing risk levels, their shortcomings are now widely recognized. Significantly, they may need to comprehensively depict the intricacy of lipid metabolism and consider changes in particle size and functionality. Consequently, there has been an increase in the pursuit of more sophisticated and all-encompassing biomarkers.

Emerging Biomarkers

Novel biomarkers, such as Lp-a and apolipoproteins, provide a more detailed and sophisticated understanding of the risk of CVD. Lp-a is a lipoprotein particle that shares a structure similar to LDL but also contains an extra apolipoprotein (a) component. Higher levels of Lp-a have repeatedly been linked to a greater likelihood of experiencing cardiovascular events, regardless of conventional risk factors [15]. The INTERHEART study, a comprehensive case-control study conducted in 52 countries, highlighted the importance of Lp-a as a reliable indicator of myocardial infarction. Apolipoproteins, such as ApoB and ApoA-I, have crucial functions in the metabolism of lipoproteins. ApoB is a constituent of lipoproteins that contribute to the development of atherosclerosis, specifically LDL. On the other hand, ApoA-I is a primary constituent of HDL. The ApoB/ApoA-I ratio, which represents the equilibrium between atherogenic and anti-atherogenic particles, has been identified as a strong indicator of cardiovascular risk. The Multi-Ethnic Study of Atherosclerosis showed that the ApoB/ApoA-I ratio better predicts coronary heart disease than standard lipid measurements [16]. In addition to Lp-a and apolipoproteins, other novel biomarkers are now being studied. Extensive research has been conducted on high-sensitivity C-reactive protein (hs-CRP), a marker used to measure systemic inflammation. Its ability to predict cardiovascular risk has been thoroughly investigated. The Justification for the Use of Statins in Primary Prevention: An Intervention Trial Evaluating Rosuvastatin (JUPITER) study showed that statin medication successfully decreased cardiovascular events in persons with high hs-CRP but normal LDL-C levels, emphasizing the potential usefulness of targeting inflammation as a treatment approach. Genetic indicators, namely polymorphisms linked to lipid metabolism, are becoming increasingly important. GWAS has revealed genetic variations associated with lipid characteristics, providing insights into an individual's vulnerability to dyslipidemia and cardiovascular risk.

Nonetheless, incorporating genetic markers into therapeutic application necessitates meticulously examining ethical, social, and practical ramifications [16]. Advanced imaging techniques revolutionize the process of evaluating cardiovascular risk by allowing us to observe atherosclerotic lesions and directly determine plaque's fragility. Out of these different methods, using computed tomography to measure coronary artery calcium score has proven to be a reliable technique for predicting risk. Coronary artery calcification is directly related to the total severity of atherosclerosis and can independently predict the occurrence of cardiovascular events. Ultrasonography can quantify the carotid intima-media thickness (CIMT), which allows for a non-invasive evaluation of the early stages of atherosclerosis in the carotid arteries. Elevated CIMT is linked to a higher likelihood of experiencing cardiovascular events, making it a viable method for assessing risk, especially in those without symptoms [16]. Intravascular imaging modalities, such as IVUS and optical coherence tomography (OCT), provide detailed and precise visualization of coronary arteries with excellent resolution. IVUS offers valuable information on the composition and structure of plaques, helping to identify plaques that are susceptible to rupture. Due to its exceptional resolution, OCT enables the precise observation of small structures within the coronary arteries. This makes it easier to accurately identify and describe plaques and evaluate stent placement during coronary procedures. Nuclear imaging techniques, such as positron emission tomography (PET) and single-photon emission computed tomography, allow for the evaluation of blood flow and tissue viability in the heart. These modalities are essential for assessing the extent of ischemia, informing treatment choices, and predicting cardiovascular outcomes in persons with suspected or confirmed coronary artery disease [17].

Therapeutic interventions

CVDs continue to be a worldwide health problem, requiring a comprehensive strategy for treatment approaches. This essay explores therapeutic techniques, focusing on lipid-modifying drugs and lifestyle adjustments. This thorough investigation examines a wide range of tactics, including statins' fundamental role, the development of new medicines, the impact of exercise, and dietary interventions. It aims to provide insights for optimizing lipid profiles and reducing cardiovascular risk.

Lipid-Modifying Agents

Statins, also known as HMG-CoA reductase inhibitors, are the most essential drugs in the class of medicines that change lipids. Statins efficiently reduce the levels of LDL-C, the main contributor to atherosclerosis development, by preventing cholesterol production. Significant clinical trials, such as the 4S and the CTT Collaboration, definitively confirmed the cardiovascular advantages of statin therapy [17]. The processes responsible for the effectiveness of statins go beyond the decrease in LDL cholesterol. Statins demonstrate pleiotropic actions, such as anti-inflammatory, antioxidant, and endothelial-stabilizing capabilities. These additional advantages are crucial in the initial and subsequent prevention of CVDs. The selection of a statin and its dosage is determined by the specific characteristics of each patient, with a preference for high-intensity statin therapy for individuals at a heightened risk of cardiovascular events. In addition, the 2018 guidelines for cholesterol management highlight the importance of tailoring the strategy to each patient, taking into account their preferences, any medical conditions they may have, and any potential interactions between other medications they are taking [17]. Although statins have been successful, there is still a remaining risk of cardiovascular problems. This has led to the investigation of other medicines that can change lipids. Fibrates, a class of drugs, principally focus on regulating triglyceride metabolism and promoting an increase in HDL-C. Fenofibrate, gemfibrozil, and bezafibrate are often prescribed fibrates. They stimulate peroxisome proliferator-activated receptors, affecting the expression of lipid metabolism genes. Fibrates lower triglyceride levels, raise HDL cholesterol, and may slightly impact LDL cholesterol. The Action to Control Cardiovascular Risk in Type 2 Diabetes (ACCORD) trial and the Fenofibrate Intervention and Event Lowering in Diabetes (FIELD) investigation examined the impact of fibrates on cardiovascular risk in patients with diabetes, revealing diverse implications on cardiovascular outcomes [18]. Although fibrates are generally well-tolerated, their usage necessitates meticulous evaluation of unique patient attributes and any medication interactions. Ongoing research seeks to identify distinct patient groups who may experience the most significant advantages from fibrates, clarifying their function in cardiovascular treatment. Proprotein convertase subtilisin/kexin type 9 (PCSK9) inhibitors are a groundbreaking addition to the medicines that change lipids. Monoclonal antibodies like evolocumab and alirocumab specifically focus on PCSK9, a protein that breaks down LDLR. This action enhances the liver's ability to eliminate LDL from the bloodstream. Clinical trials, such as the Further cardiovascular OUtcomes Research with PCSK9 Inhibition subjects with Elevated Risk (FOURIER) study and the ODYSSEY Outcomes trial, have shown the impressive effectiveness of PCSK9 inhibitors in lowering LDL-C levels and preventing cardiovascular events [18,19]. These medicines are especially beneficial for those with FH or those who have difficulty tolerating statins. Nevertheless, the excessive expense associated with PCSK9 inhibitors impedes their extensive implementation. Ongoing endeavors are being made to tackle the cost-effectiveness and broaden the availability of these powerful drugs that lower lipids. These efforts have the potential to alter the field of cardiovascular prevention significantly. The advancing comprehension of lipid metabolism and atherosclerosis has stimulated the investigation of innovative treatment pathways. Inclisiran, a siRNA specifically targeting the manufacture of PCSK9, is a novel and inventive method. The RadiO fRequency ablatION for hemorrhoids (ORION) trials exhibited a long-lasting decrease in LDL-C levels by biennial administration, highlighting the possibility of alternate pathways in lipid control [19]. Moreover, ongoing studies are being conducted on apoB antisense oligonucleotides, angiopoietin-like 3 inhibitors, and other new targets. These experimental treatments show potential for patients with treatment-resistant dyslipidemia or those who cannot tolerate current lipid-modifying medications, signaling a new era in personalized therapy for cardiovascular well-being.

Lifestyle Modifications and Dietary Interventions

Regular physical activity is crucial to maintaining cardiovascular health and should be incorporated into one's lifestyle. Physical activity has a diverse impact on how our body processes fats, leading to a beneficial lipid profile and reducing the risk of CVD. Aerobic exercise, which involves continuous and rhythmic physical activity, has been demonstrated to elevate HDL-C levels and enhance lipid metabolism. For optimal cardiovascular health, the American Heart Association advises engaging in a minimum of 150 minutes of moderate-intensity activity or 75 minutes of vigorous-intensity exercise every week [19]. Resistance training, which emphasizes muscle strength and endurance, enhances lipid profiles when combined with aerobic activity. Aerobic and resistance exercise integration has been linked to enhancements in LDL-C, HDL-C, and triglyceride levels. Engaging in regular physical exercise boosts the functioning of the endothelium, improves the body's ability to respond to insulin, and aids in the management of weight. These beneficial benefits collectively contribute to a decrease in total cardiovascular risk. High-intensity interval training (HIIT) is a form of exercise that involves short intervals of intensive activity followed by periods of relaxation. It has become popular due to its time-efficient cardiovascular advantages. HIIT has demonstrated positive effects on lipid profiles, insulin sensitivity, and cardiorespiratory fitness, as supported by research [20]. Maintaining a consistent and active lifestyle is crucial for long-term cardiovascular advantages. Including pleasurable activities, diversifying exercise regimens, and incorporating physical activity into daily routines all contribute to sustained adherence over a prolonged period. Behavioral interventions, such as creating goals and providing social support, are essential for promoting a physically active lifestyle. Although the advantages of physical activity are widely recognized, obstacles to its acceptance and maintenance continue to exist. Obstacles encompass limitations in time availability, absence of drive, and external circumstances. Customizing exercise prescriptions based on individual preferences and capabilities and implementing behavioral interventions increases the probability of maintaining adherence over time. Additionally, specific concerns are relevant for those with cardiovascular ailments or restricted physical mobility. Supervised exercise programs and collaboration with healthcare specialists are necessary to guarantee the secure and efficient incorporation of physical activity into the comprehensive management plan.

Dietary Approaches to Improve Lipid Profiles

Dietary interventions are crucial for improving lipid profiles and decreasing cardiovascular risk. Lipid metabolism is greatly influenced by the content of dietary fats, the types of carbohydrates consumed, and the overall dietary patterns. The Mediterranean diet, defined by a substantial intake of fruits, vegetables, whole grains, and olive oil, has been identified as a dietary pattern promoting heart health. The Mediterranean diet, which is abundant in monounsaturated fats, promotes higher levels of HDL-C and decreases LDL-C oxidation. The Prevención con Dieta Mediterránea (Prevention with Mediterranean Diet) (PREDIMED) trial provided evidence of the cardiovascular advantages of this specific eating plan, which decreased significant cardiovascular incidents and deaths [21]. Plant-based diets, which prioritize the consumption of fruits, vegetables, legumes, and whole grains while reducing the intake of animal products, present an alternate strategy for promoting cardiovascular well-being. These diets have inherently low levels of saturated fats and cholesterol, which helps to promote good lipid profiles. The Adventist Health Study-2 established a correlation between plant-based diets and a decreased susceptibility to CVDs. The Dietary Approaches to Stop Hypertension (DASH) diet, first developed to control blood pressure, significantly impacts lipid profiles. The DASH diet, abundant in fruits, vegetables, low-fat dairy, and lean proteins, helps decrease LDL-C and total cholesterol levels. The entire strategy of this aligns with broader objectives for cardiovascular health [21]. The specific composition of dietary fats consumed substantially impacts lipid metabolism. Unsaturated fats, present in olive oil, avocados, and fatty fish, enhance HDL-C levels and protect the cardiovascular system. On the other hand, saturated fats, which are commonly found in red meat and full-fat dairy products, raise LDL-C levels and are linked to a higher likelihood of CVDs. Trans fats, mostly in partially hydrogenated oils, have gained attention due to their negative impact on lipid profiles. The understanding of the harmful influence of trans fat on cardiovascular health is highlighted by legal initiatives and public health campaigns aimed at reducing its consumption [22]. Nutraceuticals and functional foods, which include plant sterols, omega-3 fatty acids, and soluble fibers, provide additional treatment choices for optimizing lipid profiles. These bioactive chemicals help lower LDL cholesterol, regulate triglyceride levels, and have anti-inflammatory properties. The Reduction of Cardiovascular Events with Icosapent Ethyl-Intervention Trial (REDUCE-IT) trial highlighted the cardiovascular advantages of icosapent ethyl, a meticulously refined version of omega-3 fatty acid, in persons with increased triglycerides despite receiving statin medication [17]. Plant sterols, present in specific margarine and fortified meals, effectively hinder cholesterol absorption, decreasing LDL-C levels [23].

Precision medicine in cardiovascular health

Progress in genetic and molecular profiling has facilitated the development of precision medicine in cardiovascular health. This essay examines the profound influence of precision medicine, specifically highlighting the significance of genetic and molecular profiling in customizing therapeutic approaches. This comprehensive research explores the complexities of a paradigm shift in cardiovascular care, from personalized medicine to its impact on therapy optimization. This shift has the potential to transform the field.

Genetic and Molecular Profiling

The advancement of precision medicine in cardiovascular health is driven by genomic discoveries that reveal the complex interaction between genetic elements and cardiovascular risk. GWAS has discovered numerous genetic variations linked to various aspects of cardiovascular physiology, such as lipid metabolism, blood pressure regulation, and thrombosis [23]. Incorporating genetic data into risk assessment signifies transitioning from a uniform approach to individualized medicine. Pharmacogenomics, an essential component of precision medicine, centers on comprehending the impact of genetic differences on drug reactions. Cardiovascular therapies involve customizing pharmaceutical regimens according to individual genetic profiles to maximize effectiveness while avoiding adverse side effects. Warfarin, a commonly prescribed medication that prevents blood clotting, is a prime example of the influence of pharmacogenomics. Specific genotypes of individuals are affected by genetic variations in the CYP2C9 and VKORC1 genes, which in turn impact the metabolism and sensitivity of warfarin. As a result, dosage modifications are required. The incorporation of genetic testing into the administration of warfarin demonstrates the capacity for personalized dosage to improve treatment results. Likewise, clopidogrel, a medication that prevents blood clotting and is often prescribed when a coronary stent is inserted, is metabolized in the liver through a process facilitated by the CYP2C19 enzyme. Genetic variations affecting the activity of CYP2C19 contribute to the variability in how individuals respond to clopidogrel, which in turn affects their risk of experiencing cardiovascular events again [24]. Identifying CYP2C19 genotypes helps determine appropriate alternative antiplatelet treatments for patients with a higher genetic susceptibility. In addition to anticoagulants and antiplatelet medicines, pharmacogenomic factors also apply to lipid-modifying drugs. Genetic differences in genes like SLCO1B1 play a role in the diversity of LDL-C decrease in response to statin treatment. Comprehending the genetic foundation of statin response allows customized prescriptions to maximize the equilibrium between effectiveness and tolerability. FH is an inherited condition characterized by high LDL cholesterol levels. It is a prime example of how precision medicine can be applied in cardiovascular care. FH is caused by genetic abnormalities in the genes that encode LDLR, APOB, and PCSK9. These mutations lead to early and severe atherosclerosis, a condition characterized by plaque buildup in the arteries [25]. Cascade screening among family members enables the detection of individuals with FH, facilitating timely intervention and preventive actions. Statins, commonly used as the initial treatment, can be supplemented with additional medications that affect lipid levels based on individual risk factors. Evolocumab and alirocumab, PCSK9 inhibitors, offer new therapy options for individuals with FH, demonstrating the incorporation of genetic knowledge into treatment choices [25].

Implications for Treatment Optimization

Precision medicine is now being applied to percutaneous coronary intervention (PCI), focusing on optimizing customized antiplatelet treatment. The choice of P2Y12 inhibitors, such as clopidogrel, prasugrel, or ticagrelor, is determined by hereditary variables that affect how the drugs are broken down and how the body responds to them. The TRITON-TIMI 38 trial showed that prasugrel is more effective than clopidogrel in lowering cardiovascular events in those who undergo PCI, especially in those who do not have CYP2C19 loss-of-function alleles [26]. On the other hand, people who have CYP2C19 loss-of-function alleles experience decreased effectiveness of clopidogrel and might find it advantageous to use other P2Y12 inhibitors, including ticagrelor or prasugrel. In conjunction with platelet function testing, genetic testing to detect CYP2C19 variations enables the customization of antiplatelet medication based on individual responsiveness. This precise technique reduces the likelihood of recurring cardiovascular events while also minimizing the possibility of bleeding issues that are linked to enhanced antiplatelet treatments. Genetic risk scores include many genetic variations linked to cardiovascular risk, providing a comprehensive assessment method. The scores thoroughly assess an individual's susceptibility to cardiovascular illnesses, informing medical treatment choices and lifestyle modifications. The Polygenic Risk Score for Coronary Artery Disease (PRS-CAD), calculated based on GWAS data, classifies individuals into three genetic risk categories: low, moderate, and high [27]. Using the PRS-CAD in clinical risk assessment improves the accuracy of risk prediction, identifying individuals who may gain advantages from more intensive risk reduction methods. Lipid management in precision medicine goes beyond pharmacogenomics to include broader genetic factors that affect lipid metabolism. Genetic variations linked to increased LDL-C or decreased HDL-C provide individualized strategies for reducing lipid levels. Patients diagnosed with familial mixed hyperlipidemia, a condition marked by increased levels of both LDL-C and triglycerides, could potentially see positive outcomes from a treatment approach that focuses on various lipid pathways. Understanding the genetic causes of dyslipidemia helps choose the most effective lipid-modifying drugs, leading to better treatment results.

Challenges and Considerations in Implementation

Although the concept of precision medicine is appealing, obstacles to its broad implementation still need to be addressed. To include genetic and molecular profiling in regular clinical practice, it is necessary to tackle challenges concerning the expense, availability, and comprehension of intricate genetic information. Although genetic testing is becoming more widely accessible, it may not be universally available or affordable. It is imperative to make concerted efforts to decrease expenses and broaden the availability of genetic testing to guarantee fair and equal access to precision medicine. Furthermore, understanding genetic information requires the cooperation of genetic counselors, doctors, and individuals to put the results into context and guide decision-making. The significance of solid policies and protections is emphasized by ethical considerations, particularly regarding privacy issues associated with genetic data. Ensuring transparency in communicating genetic risk information is crucial for promoting informed decision-making and building confidence between healthcare practitioners and patients. Continuing education and training for healthcare professionals is necessary to incorporate precision medicine into cardiovascular care. Comprehending the subtle distinctions in genetic and molecular profiling, as well as the practical utilization of this knowledge in clinical decision-making, is crucial for the effective execution of precision medicine.

## Conclusions

This comprehensive review sheds light on the complex relationship between genetic, molecular, and lifestyle variables influencing cardiovascular health by summarizing the current viewpoints on lipid diseases and cardiovascular risk. The synthesis highlights the numerous mechanisms involved in lipid metabolism and the dynamic nature of treatment interventions. It emphasizes the complexity of the topic and the importance of employing nuanced and tailored approaches. The consequences for clinical practice are significant. The introduction of precision medicine, which focuses on genetic and molecular profiling, requires a fundamental change in how therapeutic decisions are made. The customization of therapies based on individual risk profiles, considering pharmacogenomic factors, and adding lifestyle adjustments highlight the progression toward individualized cardiovascular care. Clinicians must skillfully traverse this field, utilizing progress to enhance patient results while tackling issues about availability, understanding, and ethical concerns. As we journey into the next phase of cardiovascular research, clear recommendations for future efforts emerge. Further investigation into genetic markers, molecular pathways, and the incorporation of new technologies will enhance our comprehension of cardiovascular etiology. Comprehensive and long-term research is necessary to confirm the effectiveness of precision medicine methods in various populations. Furthermore, it is essential to have interdisciplinary cooperation among physicians, geneticists, and researchers to advance precision medicine from a theoretical concept to practical implementation. By cultivating a solid basis of knowledge and converting it into empirical clinical observations, we create the conditions for a revolutionary period in cardiovascular well-being.
